# Variation in the Lipin 1 Gene Is Associated with Birth Weight and Selected Carcass Traits in New Zealand Romney Sheep

**DOI:** 10.3390/ani10020237

**Published:** 2020-02-03

**Authors:** Guan Wang, Huitong Zhou, Hua Gong, Jianning He, Yuzhu Luo, Jon G. H. Hickford, Jiang Hu, Jiqing Wang, Xiu Liu, Shaobin Li

**Affiliations:** 1 Gansu Key Laboratory of Herbivorous Animal Biotechnology, Faculty of Animal Science and Technology, Gansu Agricultural University, Lanzhou 730070, China; 2 International Wool Research Institute, Gansu Agricultural University, Lanzhou 730070, China; 3 Gene-Marker Laboratory, Faculty of Agriculture and Life Sciences, Lincoln University, Lincoln 7647, New Zealand; 4 College of Animal Science and Technology, Qingdao Agricultural University, Qingdao 266109, China

**Keywords:** Lipin 1 gene (*LPIN1*), nucleotide variation, PCR-SSCP, birth weight, meat yield

## Abstract

**Simple Summary:**

Lipin 1 plays an important role in lipid metabolism. It not only acts as a phosphatidate phosphatase and directly participates in the synthesis of glycerol and osteolipid, but also acts as a transcriptional co-activator to indirectly regulate the expression of genes related to lipid metabolism. In livestock species, variation in the lipin 1 gene (*LPIN1*) has been reported in pigs, chickens, and dairy cows, but has not been investigated in sheep, and little is known about whether it might affect production traits in this globally important meat-producing species. In this study, we used polymerase chain reaction-single strand conformation polymorphism (PCR-SSCP) analyses to search for variation in ovine *LPIN1*, and its effects on production and carcass traits were investigated in New Zealand Romney sheep. The results suggest that ovine *LPIN1* is variable and it may have value as a genetic marker for improving meat production and carcass traits.

**Abstract:**

Lipin 1 plays an important role in lipid metabolism. In this study; we searched for variation in the ovine lipin 1 gene (*LPIN1*) in three gene regions (a 5′ non-coding region; a region containing an alternatively spliced exon in intron 4; and a region containing coding exon 6) using polymerase chain reaction-single strand conformation polymorphism (PCR-SSCP) analysis. The greatest amount of alleles was found in coding exon 6; with five sequences being detected. The effect of variation in this exon was investigated in 242 New Zealand Romney lambs derived from 12 sire-lines. The presence of variant *E*_3_ was associated with a decrease in birth weight (*p* = 0.005) and the proportion of leg yield (*p* = 0.045), but with an increase in hot carcass weight (*p* = 0.032) and the proportion of loin yield (*p* = 0.014). The presence of variant *B*_3_ was associated with an increased pre-weaning growth rate (*p* = 0.041), whereas the presence of variant *C*_3_ was associated with an increase in shoulder yield (*p* < 0.001). These results suggest that ovine *LPIN1* variation may have value as a genetic marker for improving meat production and carcass traits.

## 1. Introduction

Lipin 1 was first identified from the fatty liver dystrophy (fld) mouse [1]. It is a member of the lipin family of proteins, and it is involved in animal lipid metabolism and its regulation. Lipin 1 is normally present in adipose tissue, skeletal muscle and heart tissue [1,2]. It has been demonstrated that lipin 1 plays an important role in the nucleus, both through involvement in the regulation of peroxisome proliferator activated receptor gamma (PPARG)-mediated increases in adipogenic gene expression, and as an enzyme in the triglyceride synthesis pathway [3]. Increased lipin 1 expression in either adipose tissue or skeletal muscle results in increased adiposity [4]. Conversely, a lack of lipin 1 results in the metabolic abnormalities observed in fld mice [1]. 

Two major isoforms of lipin 1 (lipin 1α and lipin 1β) have been reported in humans [5]. Lipin 1α is predominantly active in the nucleus and plays a role in differentiation, whereas lipin 1β is primarily located in the cytoplasm of adipocytes and is predominantly involved in lipogenesis. This suggests that these two isoforms have distinct but complementary functions [5]. Lipin 1β differs from lipin 1α in that it is produced from a transcript that contains an alternatively-spliced exon of 99-bp, which is located in intron 4. This results in the in-frame addition of 33 amino acids to the mature protein [5].

Lipin 1 is associated with some diseases other than lipodystrophy in humans. Mutations in the lipin 1 gene (*LPIN1*) are a major cause of severe rhabdomyolysis in early childhood, and the heterozygous carrier state may predispose one to statin-induced myopathy [6,7]. Single nucleotide changes in *LPIN1* have also been found to be associated with serum insulin levels and body mass index in dyslipidaemic Finnish families [8], and a common variation in *LPIN1* has been reported to be related to body mass index in the UK population [9].

In livestock species, variation in *LPIN1* has been reported in pigs, chickens, and dairy cows [10,11,12,13]. He et al. [10] reported that *LPIN1* variation was associated with the proportion of leaf fat (the fat that lines the abdominal cavity and encloses the kidneys especially) and intramuscular fat in pigs, while Wang et al. [12] described associations between *LPIN1* variation and performance and carcass traits in chickens. In dairy cows, *LPIN1* variation was found to be associated with milk traits [11,13].

Variation in *LPIN1* has not been investigated in sheep, and hence it is not known whether it might affect production traits in this globally important meat-producing species. However, the Ensembl database (www.ensembl.org) does describe numerous nucleotide sequence variations in the 22 exons of ovine *LPIN1* (ENSOARG00000016144), based on bioinformatics analysis of ovine chromosome 3 sequences (Oar_v3.1:CM001584.1). In this context, we therefore used polymerase chain reaction-single strand conformation polymorphism (PCR-SSCP) analyses to search for variation in ovine *LPIN1* and linear modeling to ascertain its effects on production and carcass traits in New Zealand (NZ) Romney sheep.

## 2. Materials and Methods

All research involving animals was carried out in accordance with the Animal Welfare Act 1999 (NZ Government), and the procedure of blood sample collection used in this study comes under the umbrella of normal farming practice. The method is similar to earmarking and is covered by Section 7.5 Animal Identification of the Animal Welfare (Sheep and Beef Cattle) Code of Welfare 2010, a code of welfare issued under the Act. 

### 2.1. Sheep Investigated and Data Collection

To screen for variation in ovine *LPIN1*, fifty sheep from twenty-five different farms with NZ Romney, Merino, or White Dorper sheep and crosses were screened. Subsequently, 242 NZ Romney lambs, the progeny of 12 independent sire-lines, were used for the association study. All of these lambs were born and reared on the same farm, and their birth date, birth rank (i.e., whether they were a single, twin or triplet), rearing-rank (i.e., whether they were raised as a single, twin or triplet), birth weight, and gender were recorded. The lambs were weaned at approximately 12 weeks of age, and the pre-weaning growth rates were calculated in grams per day (g/day). All male lambs and some ewe lambs were drafted for slaughter, and draft age and draft weight were recorded for each lamb.

For each lamb slaughtered, hot carcass weight (HCW) was measured on the processing chain (Alliance Food Limited, Smithfield, Timaru, New Zealand). Video image analysis (VIAScan, Sastek, Australia), developed by Meat and Livestock Australia and described in Hopkins [14], was used to estimate the following traits: V-GR (fat depth at the 12th rib), meat yield (expressed as a percentage of HCW) in the leg (called leg yield), loin (loin yield) and shoulder (shoulder yield), total meat yield (the sum of the leg, loin and shoulder yields for any given carcass), the proportion of leg yield, the proportion of loin yield, and the proportion of shoulder yield. The proportion yield of leg, loin or shoulder is the yield of the specific area divided by the total yield, and is expressed as a percentage. 

### 2.2. Blood Samples and Polymerase Chain Reaction (PCR) Amplification

Blood samples were collected from each sheep onto TFN paper (Munktell Filter AB, Sweden), and genomic DNA used for PCR amplification was purified from the dried blood spot using a two-step procedure described by Zhou et al. [15]. 

Primers (Table 1) were designed to amplify three regions of ovine *LPIN1* based on the sequence ENSOARG00000016144. These three regions were: a 5′ non-coding region, a region that has been revealed to contain an alternatively spliced exon in *LPIN1β* (called the *LPIN1β*-spliced exon here), and a region spanning the 319-bp coding exon 6 (one of the larger coding exons). These primers were synthesized by Integrated DNA Technologies (Coralville, IA, USA). PCR amplifications were performed in a 15-μL reaction containing the DNA on one punch of TFN paper, 150 μM of each deoxyribonucleoside (dNTP) (Bioline, London, UK), 0.25 μM of each primer, 0.5 U of Taq DNA polymerase (Qiagen, Hilden, Germany), 2.5 mM Mg^2+^, and 1× reaction buffer supplied with the enzyme and ddH_2_O to make up the volume. The thermal profile consisted of 2 min at 94 °C, followed by 35 cycles of 30 s at 94 °C, 30 s at 60 °C, and 30 s at 72 °C, with a final extension of 5 min at 72 °C.

### 2.3. Screening for Sequence Variation

The PCR amplicons were screened for sequence variation using SSCP analysis. Each amplicon (0.7 μL) was mixed with 7 μL of loading dye [98% formamide, 10 mM ethylenediaminetetraacetic acid (EDTA), 0.025% bromophenol blue, and 0.025% xylene cyanol]. After denaturation at 95 °C for 5 min, the samples were rapidly cooled on wet ice and then electrophoresed in 16 cm × 18 cm, acrylamide:bisacrylamide (37.5:1) (Bio-Rad) gels in 0.5 × Tris-borate-EDTA (TBE) buffer at 25 °C, 350 V for 19 h. The gels were silver-stained according to the method of Byun et al. [16].

### 2.4. Sequencing of the Variants and Sequence Analysis

PCR amplicons representing different SSCP banding patterns from sheep that appeared to be homozygous were sequenced using Sanger sequencing in both directions at the Lincoln University DNA sequencing facility, New Zealand. Variants that were only found in heterozygous sheep were sequenced using an approach described by Gong et al. [17]. Briefly, a band corresponding to the variant was cut as a gel slice from the polyacrylamide gel, macerated, and then used as a template for re-amplification with the original primers. This second amplicon was then sequenced directly, as described above for the homozygous patterns.

Nucleotide sequence alignments and translation to amino acid sequences were undertaken using DNAMAN (version 5.2.10, Lynnon BioSoft, Vaudreuil, QC, Canada).

### 2.5. Statistical Analyses

All analyses were performed using MINITAB version 16 (State College, PA, USA). Generalized linear mixed models (GLMMs) were first used to assess the effect of the presence or absence (coded as 1 or 0, respectively) of individual variants on growth traits (including birth weight and growth rate) and the various carcass traits (including V-GR, leg yield, loin yield, shoulder yield, total lean-meat yield, and proportion of leg yield, loin yield and shoulder yield). Those variants that had associations with a *p*-value of less than 0.2 (*p* < 0.20) and that could thus potentially impact the trait were subsequently factored into the models, such that we could determine the independent variant effects.

Sire and gender were fitted as random and fixed factors, respectively, in the GLMMs, and age was fitted as a random factor for all traits, except for birth weight and pre-weaning growth rate. All of the traits were corrected for either birth weight, birth rank or rearing-rank, depending on which had the largest effect on the trait.

Unless otherwise indicated, all *p*-values were considered statistically significant when *p* < 0.05, and trends were noted at *p* < 0.10.

## 3. Results

### 3.1. Variation in Ovine LPIN1

**Figure 1:**
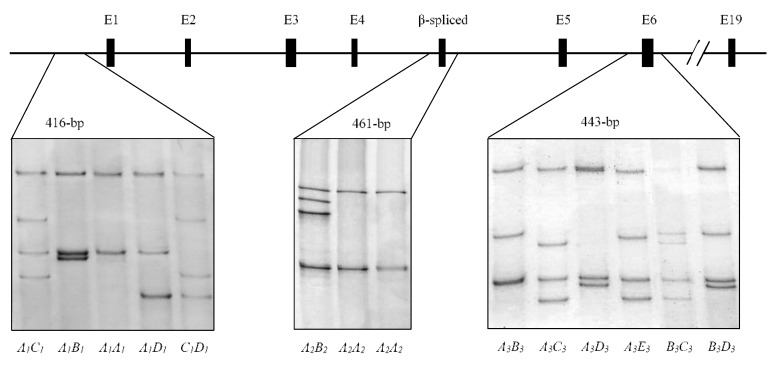
Sequence variation in the ovine lipin 1 gene (*LPIN1*) identified by polymerase chain reaction-single strand conformation polymorphism (PCR-SSCP) analysis. Different SSCP banding patterns for amplicons from the three gene regions are shown in either homozygous or heterozygous forms. “E” indicates a coding exon, and “β-spliced” indicates the alternatively spliced exon used in *LPIN1β*.

Four PCR-SSCP banding patterns were detected in the 5′ non-coding region, two banding patterns were detected in the *LPIN1β*-spliced exon region, and five banding patterns were found in the coding exon 6 region (Figure 1). DNA sequencing revealed that these PCR-SSCP patterns represented four, two and five variant sequences, respectively (named *A*_1_, *B*_1_, *C*_1_ and *D*_1_; *A*_2_ and *B*_2_; and *A*_3_, *B*_3_, *C*_3_, *D*_3_ and *E*_3_). These sequences were deposited into the GenBank with accession numbers MN548886 to MN548896, respectively.

The 5′ non-coding region variants *A*_1_, *B*_1_, *C*_1_ and *D*_1_ were detected at frequencies of 58.0%, 16.0%, 12.0% and 14.0%, respectively. In the *LPIN1β*-spliced exon region, variant *A*_2_ was most common with a frequency of 93.0%, while variant *B*_2_ occurred only at 7.0%. For the coding exon 6 region, the frequencies of variants *A*_3_, *B*_3_, *C*_3_, *D*_3_ and *E*_3_ were 18.0%, 24.0%, 29.0%, 19.0% and 10.0%, respectively.

Six nucleotide substitutions were found in the 5′ non-coding region, and these were c.-31280A/G, c.-31290C/T, c.-31449C/T, c.-31475A/G, c.-31539C/T and c.-31556C/T. One synonymous substitution (c.722+1965A/G) was found in the *LPIN1β*-spliced exon. This is identified as rs424786364 in the Ensembl database.

**Figure 2:**
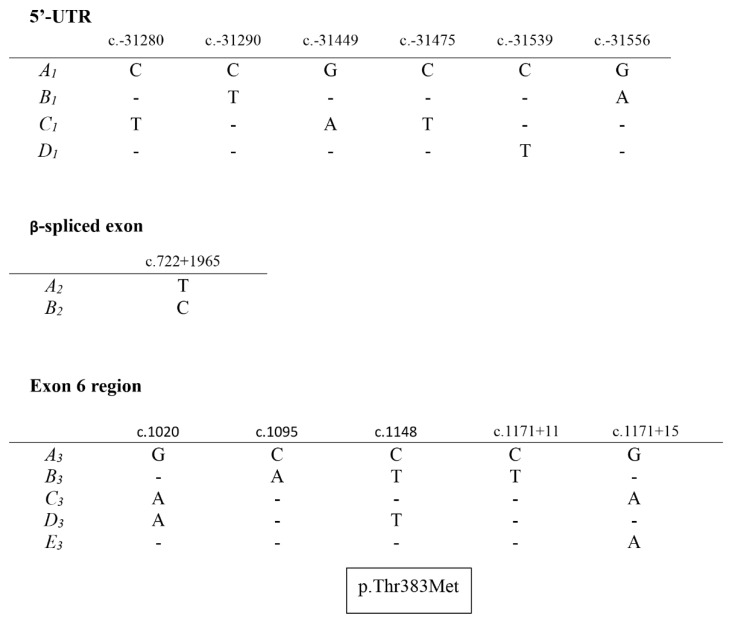
Variant sequences identified in three regions of ovine *LPIN1*. Only the nucleotide differences are shown, and dashes represent nucleotide sequences identical to the top sequence. The numbering of nucleotides follows the Human Genome Variation Society (HGVS) recommended nomenclature (http://varnomen.hgvs.org/). The non-synonymous substitution in coding exon 6 is indicated along with the putative amino acid change.

Five substitutions were detected in the coding exon 6 region including two synonymous substitutions (c.1020A/G: rs424526247 and c.1095A/C: rs4059700209), a non-synonymous substitution (c.1148C/T: rs402994834) and two substitutions (c.1171+11C/T: rs418585403 and c.1171+15A/G: rs429800809) in intron 6 (Figure 2). It should be noted that Ensembl allocates the latter two nucleotide sequence variations to exon 6 when analysis of this region of the gene sequence suggests that these are intron sequences. The non-synonymous substitution (rs402994834) would lead to a p.Thr383Met amino acid substitution, if translated.

### 3.2. Effect of LPIN1 Variation on Production Traits

Of the three gene regions investigated, the coding exon 6 region was most variable, and hence the effect of variation in this region on the various production traits was investigated in 242 NZ Romney lambs. The average productions traits for individual *LPIN1* genotypes are shown in Table S1, together with the carcass traits. Variant *B*_3_ was found to be associated with increased pre-weaning growth rate and trended towards association with an increase in birth weight and the proportion of shoulder yield, but these trends disappeared when corrected for other variants (Table 2). Variant *C*_3_ was associated with increased shoulder yield and decreased proportion of loin yield, and the association with shoulder yield became more significant when corrected for the other variants. The association of variant *C*_3_ with proportion of loin yield was lost when corrected for other variants (Table 2). The presence of variant *E*_3_ was found to be associated with a decrease in birth weight and proportion of leg yield, and an increase in hot carcass weight (Table 2). Variant *E*_3_ trended towards association with an increase in fat depth at the 12th rib and proportion of loin yield, and this became significant when corrected for other variants (Table 2).

## 4. Discussion

This study explored genetic variation in three regions of ovine *LPIN1*, and the effect of variation in coding exon 6 was investigated on some key growth and carcass traits in NZ Romney sheep. Sequence variation was found in all of the regions, with each having two to five sequence variants. This confirms that ovine *LPIN1* is variable and suggests that further investigation of *LPIN1* variation in different sheep breeds is worthwhile, especially as only a small number of sheep from a limited number of breeds were investigated in this study. It is likely that more new variants will be found when more samples and different sheep breeds are investigated.

Variation detected in ovine *LPIN1* included synonymous and non-synonymous nucleotide substitutions in coding regions, and nucleotide substitutions in the 5′ non-coding region and introns. The non-synonymous substitution detected in coding exon 6 would lead to a threonine to methionine amino acid change at a position where the threonine residue has been found to be highly conserved across mammalian species, with a methionine residue only being reported in two other species: the northern sea otter (*Enhydra lutris kenyoni*) (GenBank XP_022369157–XP_022369159) and the wolverine (*Gulo gulo*) (GenBank VCW69084). This non-synonymous nucleotide substitution may affect protein structure and consequently the function of the protein. The nucleotide substitutions found in the 5′ non-coding region and introns may affect gene expression, and sequence variation can also affect translation rates and co-translational protein folding [18].

In lamb production systems, birth weight is considered to be an important factor in determining the growth potential of a lamb and it is related to the mature body weight potential of sheep [19]. Although higher birth weight lambs typically have increased perinatal vigor, birthing difficulties (dystocia) have been reported to increase with heavier lambs [20,21]. In the study of Gourt et al. [22], lambs with birth weights between 4.0 and 5.5 kg had good survival rates, while ewes with lambs over 5.5 kg at birth were more likely to be affected by dystocia. In the present study, it was notable that the average birth weight of these living NZ Romney lambs was 6.13 ± 0.99 kg, with very low levels of perinatal mortality (data not shown), this suggesting that the upper threshold for birthweight suggested by Gourt et al. [22] can be exceeded without dystocia commonly occurring. In the present study, lambs with the *E*_3_ variant were found to have lower mean birth weights (5.7 ± 0.14 kg), which is 0.4 kg less than the lambs that did not carry this variant (6.1 ± 0.11 kg). However, the presence of variant *E*_3_ did not result in a loss of HCW and total lean meat yield in these NZ Romney lambs. Instead, lambs with *E*_3_ produced higher HCW than lambs that did not contain variant *E*_3_. Given that total lean meat yield is expressed as a percentage of HCW, with the same total lean yield, lambs with higher HCWs will have more lean meat than lambs with lower HCWs. This suggests that lambs with variant *E*_3_ produced more lean meat than lambs without variant *E*_3_, which may in part contribute to the increase in HCW associated with *E*_3_. Furthermore, lambs carrying variant *E*_3_ had a lower proportion of leg yield, but a higher proportion of loin yield, compared to lambs that did not carry *E*_3_. This may have a commercial benefit because of its location in the carcass. The loin is known to be a high-priced ‘bone-out’ cut of lamb and is known as a ‘backstrap’, while bone-in is part of the ‘Frenched-rack’.

Fat deposition may also contribute to the increase in HCW seen for variant *E*_3_, which is supported by the finding that this variant was trending towards association with increased fat depth at the 12th rib.

Overall, these results suggest that variation in *LPIN1* affects birth weight and muscle growth traits. Selection for variant *E*_3_ may have potential to result in lower birth weights, but more lean meat with a high proportion of loin meat, while selection for *C*_3_ could lead to a high proportion of shoulder meat.

## Figures and Tables

**Table 1:** Polymerase Chain Reaction (PCR) primers used for amplification of three regions of ovine *LPIN1*.

Gene Region	Primer Sequence (5’–3’)	Amplicon Size (Bp)
5′ non-coding region	F: ACAAGGAGAGAACATGGGAG	416
	R: CACACCTCAGCACTGGGTC	
*LPIN1β*-spliced exon	F: AGCAATTCATTATGGGCCTGC	461
	R: CACATAAGTAATTTGGTTAATGG	
Coding exon 6	F: GATCCAGTCCTCACCACAC	443
	R: CAAGAGAGATGTCCTGTCTC	

**Table 2:** Associations between the presence/absence of *LIPIN1* variants and variation in selected production traits.

Trait	Variant Assessed ^2^	Other Variants Fitted	Mean ± SE ^3^		*p*-Value
			Absent	Present	
Birth weight (kg)	*A*_3_	None	5.8 ± 0.11	5.9 ± 0.13	0.170
	*B*_3_	None	*5.8 ± 0.11*	*6.0 ± 0.14*	*0.061*
	*C*_3_	None	5.9 ± 0.11	5.8 ± 0.13	0.766
	*D*_3_	None	5.8 ± 0.10	5.8 ± 0.15	0.872
	*E*_3_	None	**6.0 ± 0.11**	**5.6 ± 0.13**	**0.001**
	*B*_3_	*A*_3_, *E*_3_	5.8 ± 0.10	6.0 ± 0.15	0.115
	*E*_3_	*A*_3_, *B*_3_	**6.1 ± 0.11**	**5.7 ± 0.14**	**0.005**
Pre-weaning growth rate (g/day)	*A*_3_	None	335.6 ± 6.02	333.8 ± 6.48	0.760
	*B*_3_	None	**330.9 ± 5.77**	**344.5 ± 7.20**	**0.041**
	*C*_3_	None	334.8 ± 5.97	334.9 ± 6.59	0.984
	*D*_3_	None	335.6 ± 5.56	329.5 ± 8.20	0.379
	*E*_3_	None	334.9 ± 5.99	334.8 ± 6.83	0.997
Hot carcass weight (kg)	*A*_3_	None	17.4 ± 0.28	17.5 ± 0.30	0.547
	*B*_3_	None	17.4 ± 0.27	17.6 ± 0.33	0.308
	*C*_3_	None	17.4 ± 0.28	17.4 ± 0.30	0.913
	*D*_3_	None	17.5 ± 0.26	17.2 ± 0.37	0.329
	*E*_3_	None	**17.2 ± 0.28**	**17.7 ± 0.29**	**0.032**
V-GR (mm) ^1^	*A*_3_	None	7.9 ± 0.40	7.7 ± 0.44	0.605
	*B*_3_	None	7.8 ± 0.39	8.0 ± 0.48	0.535
	*C*_3_	None	7.9 ± 0.40	7.8 ± 0.43	0.806
	*D*_3_	None	7.8 ± 0.38	7.8 ± 0.54	0.948
	*E*_3_	None	*7.5 ± 0.41*	*8.2 ± 0.42*	*0.050*
Leg yield (%)	*A*_3_	None	22.2 ± 0.18	22.2 ± 0.20	0.937
	*B*_3_	None	22.2 ± 0.17	22.2 ± 0.22	0.674
	*C*_3_	None	22.1 ± 0.18	22.3 ± 0.20	0.304
	*D*_3_	None	22.2 ± 0.17	21.9 ± 0.24	0.110
	*E*_3_	None	22.2 ± 0.19	22.1 ± 0.19	0.540
Loin yield (%)	*A*_3_	None	15.0 ± 0.13	15.1 ± 0.14	0.523
	*B*_3_	None	15.0 ± 0.12	15.2 ± 0.16	0.402
	*C*_3_	None	15.1 ± 0.13	15.1 ± 0.14	0.982
	*D*_3_	None	15.1 ± 0.12	14.9 ± 0.17	0.257
	*E*_3_	None	15.0 ± 0.13	15.2 ± 0.14	0.243
Shoulder yield (%)	*A*_3_	None	17.5 ± 0.15	17.3 ± 0.15	0.137
	*B*_3_	None	17.4 ± 0.14	17.3 ± 0.17	0.213
	*C*_3_	None	**17.2 ± 0.15**	**17.6 ± 0.15**	**0.005**
	*D*_3_	None	17.4 ± 0.13	17.2 ± 0.20	0.215
	*E*_3_	None	17.3 ± 0.15	17.5 ± 0.15	0.158
	*C*_3_	*A*_3_, *E*_3_	**17.3 ± 0.13**	**17.8 ± 0.14**	**<0.001**
Total lean meat yield (%)	*A*_3_	None	54.7 ± 0.33	54.6 ± 0.38	0.872
	*B*_3_	None	54.6 ± 0.32	54.7 ± 0.43	0.834
	*C*_3_	None	54.5 ± 0.34	55.0 ± 0.37	0.142
	*D*_3_	None	54.8 ± 0.32	54.2 ± 0.45	0.129
	*E*_3_	None	54.6 ± 0.36	54.7 ± 0.36	0.718
Proportion of leg yield (%)	*A*_3_	None	40.6 ± 0.15	40.7 ± 0.17	0.747
	*B*_3_	None	40.6 ± 0.15	40.7 ± 0.18	0.586
	*C*_3_	None	40.7 ± 0.15	40.6 ± 0.16	0.626
	*D*_3_	None	40.7 ± 0.14	40.6 ± 0.20	0.670
	*E*_3_	None	**40.8 ± 0.16**	**40.5 ± 0.16**	**0.045**
Proportion of loin yield (%)	*A*_3_	None	27.6 ± 0.12	27.7 ± 0.13	0.167
	*B*_3_	None	27.6 ± 0.12	27.7 ± 0.15	0.226
	*C*_3_	None	**27.7 ± 0.12**	**27.5 ± 0.13**	**0.029**
	*D*_3_	None	27.6 ± 0.11	27.7 ± 0.16	0.816
	*E*_3_	None	*27.5 ± 0.13*	*27.7 ± 0.13*	*0.069*
	*C*_3_	*A*_3_ *, E*_3_	27.5 ± 0.11	27.4 ± 0.13	0.414
	*E*_3_	*A*_3_ *, C*_3_	**27.3 ± 0.11**	**27.6 ± 0.13**	**0.014**
Proportion of shoulder yield (%)	*A*_3_	None	32.0 ± 0.19	31.8 ± 0.18	0.260
	*B*_3_	None	*31.9 ± 0.17*	*31.6 ± 0.21*	*0.087*
	*C*_3_	None	31.8 ± 0.18	32.0 ± 0.18	0.124
	*D*_3_	None	31.9 ± 0.17	32.0 ± 0.25	0.507
	*E*_3_	None	31.8 ± 0.19	31.9 ± 0.18	0.438
	*B*_3_	*C*_3_	32.0 ± 0.17	31.9 ± 0.21	0.545

^1^ A measure of fat depth at the 12th rib as determined by VIAScan analysis [14]; ^2^ A total of 242 lambs were included in the model; variants that occurred at a frequency under 5% were excluded. Variant *A*_3_ was present in 96 lambs and absent in 146 lambs, variant *B*_3_ was present in 84 lambs and absent in 158 lambs, and variant *C*_3_ was present in 101 lambs and absent in 141 lambs. Variant *D*_3_ was present in 52 lambs and absent in 190 lambs, and variant *E*_3_ was present in 75 lambs and absent in 167 lambs; ^3^ Predicted means and standard error derived from the generalized linear mixed models (GLMMs). *p* < 0.05 are in bold, while 0.05 ≤ *p* < 0.10 are italicized.

## Supplementary Materials

The following are available online at https://www.mdpi.com/2076-2615/10/2/237/s1, Table S1: Average production traits for individual *LPIN1* genotypes in the 242 New Zealand Romney lambs.
