# Supplementary material for: Variation in the Lipin 1 Gene Is Associated with Birth Weight and Selected Carcass Traits in New Zealand Romney Sheep
Source: Animals (Basel). 2020 Feb 3;10(2):237. doi: 10.3390/ani10020237 (PMC7071029; doi:10.3390/ani10020237)
Supplement: Supplementary file 1 [file animals-10-00237-s001.pdf]

Supplementary Table S1. Average production traits for individual *LPIN1* genotypes in the 242 New Zealand Romney lambs

| Trait | $A_3A_3$ n=17 | $A_3B_3$ n=19 | $A_3C_3$ n=34 | $A_3D_3$ n=11 | $A_3E_3$ n=15 | $B_3B_3$ n=15 | $B_3C_3$ n=16 | $B_3D_3$ n=14 | $B_3E_3$ n=20 | $C_3C_3$ n=20 | $C_3D_3$ n=14 | $C_3E_3$ n=17 | $D_3D_3$ n=7 | $D_3E_3$ n=9 | $E_3E_3$ n=14 |
|---|---|---|---|---|---|---|---|---|---|---|---|---|---|---|---|
| Birth weight (kg) | 6.1 | 6.4 | 6.4 | 6.4 | 5.7 | 6.4 | 6.2 | 6.4 | 6.0 | 5.8 | 6.0 | 5.5 | 6.5 | 6.0 | 6.1 |
| Pre-weaning growth rate (g/day) | 334.0 | 346.3 | 340.5 | 334.7 | 322.2 | 345.7 | 353.0 | 343.0 | 350.2 | 321.7 | 322.0 | 343.3 | 335.9 | 361.3 | 350.8 |
| Draft age (days) | 108.0 | 100.2 | 106.3 | 107.9 | 118.8 | 99.6 | 101.4 | 101.2 | 110.0 | 107.0 | 109.5 | 107.6 | 100.6 | 93.6 | 107.1 |
| Hot carcass weight (HCW: kg) | 17.7 | 17.4 | 17.7 | 17.9 | 17.5 | 17.1 | 18.1 | 17.1 | 18.5 | 16.3 | 17.1 | 17.6 | 16.8 | 18.3 | 18.4 |
| V-GR (mm) | 7.2 | 7.9 | 6.3 | 7.0 | 6.8 | 6.8 | 7.8 | 6.7 | 8.0 | 5.8 | 6.9 | 7.5 | 6.2 | 9.1 | 8.7 |
| Leg yield (% of HCW) | 22.5 | 22.0 | 22.8 | 22.6 | 22.1 | 22.0 | 22.4 | 22.7 | 22.2 | 22.3 | 22.7 | 22.4 | 22.7 | 22.1 | 22.1 |
| Loin yield (% of HCW) | 15.0 | 15.1 | 15.4 | 15.4 | 15.3 | 14.8 | 15.3 | 15.2 | 15.5 | 14.8 | 15.5 | 15.1 | 15.2 | 15.0 | 15.3 |
| Shoulder yield (% of HCW) | 17.4 | 17.1 | 18.0 | 17.8 | 17.3 | 17.4 | 17.9 | 17.3 | 17.5 | 17.4 | 17.9 | 17.7 | 17.7 | 17.5 | 17.7 |
| Total lean meat yield (% of HCW) | 54.9 | 54.2 | 56.1 | 55.9 | 54.7 | 54.2 | 55.6 | 55.2 | 55.2 | 54.6 | 56.1 | 55.2 | 55.6 | 54.6 | 55.1 |
| Proportion leg yield (%) | 41.0 | 40.6 | 40.5 | 40.4 | 40.5 | 40.6 | 40.3 | 41.1 | 40.2 | 40.9 | 40.5 | 40.5 | 40.7 | 40.4 | 40.1 |
| Proportion loin yield (%) | 27.4 | 27.9 | 27.5 | 27.6 | 27.9 | 27.2 | 27.6 | 27.6 | 28.0 | 27.1 | 27.6 | 27.4 | 27.4 | 27.5 | 27.9 |
| Proportion shoulder yield (%) | 31.6 | 31.5 | 32.0 | 31.9 | 31.6 | 32.2 | 32.1 | 31.3 | 31.7 | 31.9 | 31.9 | 32.1 | 31.9 | 32.1 | 32.1 |
